# Anticancer Activity of Tetrandrine by Inducing Apoptosis in Human Breast Cancer Cell Line MDA-MB-231 In Vivo

**DOI:** 10.1155/2020/6823520

**Published:** 2020-06-30

**Authors:** Chun-hui Wang, Jia-min Yang, Yu-bo Guo, Jing Shen, Xiao-hua Pei

**Affiliations:** ^1^The Fanshan Hospital, Beijing University of Chinese Medicine, Beijing 102488, China; ^2^Beijing Vocational College of Labour and Social Security, Beijing 100029, China; ^3^Beijing University of Chinese Medicine, Beijing 100029, China

## Abstract

Tetrandrine (TET) is an alkaloid extracted from a traditional Chinese medicinal plant. It exerts remarkable anticancer activity and induces apoptotic cell death in various human cancer cells. The present study aimed to investigate the effects of TET on the inhibition of tumor growth and the induction of apoptosis in MDA-MB-231 breast cancer in xenograft mice. Tumor weight and volume were measured. The histopathological changes in the tumor tissue were observed. Immunohistochemistry analysis of Bcl-2-associated X protein (Bax) and B-cell lymphoma/leukemia-2 (Bcl-2) was carried out. The expression of apoptosis-associated genes and proteins, such as cysteine aspartic acid-specific protease-3 (Caspase-3), Survivin, Bax, Bcl-2, BH3-interacting domain death agonist (Bid), and poly ADP-ribose polymerase (PARP), was measured by reverse transcription-polymerase chain reaction (RT-PCR) and Western blotting, respectively. TET inhibited tumor growth and induced apoptosis in TNBC cell line MDA-MB-231. The mechanism underlying this effect might be mediated by TET-upregulated Caspase-3, Bax, and Bid and downregulated by Bcl-2, Survivin, and PARP. Taken together, this study supported the fact that TET is a promising therapeutic agent for the treatment of TNBC, thereby providing experimental evidence for its use in the treatment of breast cancer.

## 1. Introduction

The incidence of breast cancer accounts for 7–10% of all malignant tumors in the body [1]. It is one of the most common tumors in females that threatens women health with increasing incidence. According to the survey in 2016, 246,660 invasive breast cancer patients were detected in the USA, of which about 1/6 deceased [2]. The incidence of breast cancer is also increasing every year in China. At present, the number of new breast cancer patients in China accounts for about 12.2% of the worldwide cases, and the number of deaths accounts for about 9.6% of the global rate [3].

Breast cancer is categorized into two types: noninvasive and invasive. Clinically, it is classified into three types: hormone receptor-positive, human epidermal growth factor receptor-2 (HER2) positive, and triple-negative breast cancer (TNBC) [4]. As an invasive breast carcinoma, TNBC is characterized by the absence of expression of the estrogen receptor (ER), progesterone receptor (PgR), and human epidermal growth factor receptor-2 (HER2) proteins that belong to basal cell-like breast cancer. Therefore, it was not eligible for hormone or anti-Her2 therapy. TNBC represents approximately 15–20% of all pathological types of breast cancers but accounts for a disproportionate number of breast cancer-related deaths constituting up to 5% of all cancer deaths annually [2, 5]. As a specific subtype of breast cancer and compared to the hormone receptor-positive breast cancers, TNBC has a high recurrence rate, strong invasiveness, and a worse prognosis, which commonly occurs in younger and obese women; the average age of onset was 53 years [6]. However, targeted treatment is yet lacking. Therefore, effective prevention and cure of breast cancer, improving the survival rate and quality of life and alleviating the burden on patients, have become major concerns worldwide.

At present, TNBC patients undergo combination therapies, consisting of surgery, radiation, chemotherapy, newly developed targeted therapy, and immunotherapy [4]. TNBC constitutes a heterogeneous group of malignancies that differ in natural history and response to treatment. Due to the lack of targeted therapy options, the standard care for TNBC remains chemotherapy. Although TNBC is the subtype with the most complete response to chemotherapy (22%), the recurrence and metastasis rate of such patients is still higher than that of non-TNBC tumors [6, 7]. Considering the malignancy of TNBC and the death rate of metastatic breast cancer, further studies are required to explore therapies or drugs in improving the outcome of this subtype of breast cancer.

Tetrandrine (TET) is a bis-benzylisoquinoline alkaloid isolated from a Chinese medicinal herb, han-fang-chi (or fen-fang-qi, *Stephania tetrandra S. Moore*) and has been used as a herbal therapy in Chinese medicine for hundreds of years. TET possesses antioxidant [8], anti-inflammatory [9], plasma glucose-lowering [10], immunosuppressive [11], antifibrotic [12], anticancer [13], antivirus [14], hypertension [15], silicosis [16], and reversal of chemotherapy drug resistance activities [17]. It also possesses remarkable antitumor activity in several types of cancers both in vitro and in vivo, including breast cancer [18, 19], liver cancer [20], colon cancer [21], leukemia [22], lung cancer [23], prostate cancer [24], and cervical cancer [25].

The primary mechanism of action of TET is related to multiple factors, such as modulating molecular signaling pathways [26, 27], inducing cancer cells apoptosis [28, 29], promoting cell cycle arrest [30, 31], and increasing cell autophagy [18]. Although TET plays a significant role of an antibreast cancer therapeutic, its effect on TNBC is to be elucidated.

In this study, the anticancer activity of TET against the TNBC cell line MDA-MB-231 was focused on apoptosis. The histopathological changes in the tumor tissue were observed, the immunohistochemistry of Bcl-2-associated X protein (Bax) and B-cell lymphoma/leukemia-2 (Bcl-2) was analyzed, and the expression of cysteine aspartic acid-specific protease-3 (Caspase-3), Survivin, Bax, Bcl-2, BH3-interacting domain death agonist (Bid), and poly ADP-ribose polymerase (PARP) genes and proteins was detected by RT-PCR and Western blotting, respectively.

## 2. Materials and Methods

### 2.1. Animals and Grouping

A total of 30 female BALB/c nude mice, aged 4 weeks weighing 16 ± 2 g (purchased from Beijing Vital River Laboratory Animal Technology Co., Ltd. (Beijing, China)), were housed in a room that was maintained at a constant temperature of 23 ± 1°C. The room was maintained at a constant humidity of 45*p*.100 ± 5*p*.100 and a 12 h : 12 h light/dark cycle with light onset at 08:00 a.m. Drinking water and laboratory rodent chow were provided *pour ad libitum*. All studies were conducted in the facilities of the Beijing University of Chinese Medicine and approved by the Medical and Experimental Animal Ethics Committee of Beijing University of Chinese Medicine (no. BUCM-4-2017122028-4028).

### 2.2. Medicine

Capecitabine was purchased from Shanghai Roche Pharmaceutical Co., Ltd. (Shanghai, China). TET was purchased from Zhejiang Jinhua CONBA Biopharm. Co., Ltd. (Jinhua, Zhejiang, China).

### 2.3. Reagents and Apparatus

Mouse anti-Bax, mouse anti-Bcl-2, rabbit anti-Caspase-3, rabbit anti-Bid, rabbit anti-PARP, rabbit anti-Survivin, and rabbit anti-GAPDH were obtained from Proteintech (USA). Horseradish peroxidase- (HRP-) conjugated goat anti-mouse and anti-rabbit IgG antibodies were provided by Beijing Zhongshan Golden Bridge Biotechnology Co. Ltd. (Beijing, China). Protein assay kit was obtained from Beijing Pulilai Gene Technology Co., Ltd. (Beijing, China). Reverse transcription kit was obtained from Promega (USA).

### 2.4. Medicinal Dosage and Administration

The clinical dosage conversion between humans and mice was estimated based on a previous study: human medicinal dosage (g/kg): mice medicinal dosage (g/kg) = 12.33 : 1 [32]. If a person (60 kg) receives 10 g of each medicine, the equivalent dose for a mouse will be 2.055 g/kg. The daily dosage of intragastric administration for each nude mouse was 1 mg for capecitabine and 2 mg for TET. Capecitabine and TET were prepared as 10 mg/mL and 20 mg/mL suspensions in sterile deionized water, respectively.

### 2.5. Cell Culture

Human breast cancer cell MDA-MB-231 was obtained from Cell Resource Center, Shanghai Institutes for Biological Sciences, Chinese Academy of Sciences and cultured in Dulbecco's modified Eagle's medium (DMEM) supplemented with 10% fetal bovine serum (FBS) (Gibco, China), 100 U/mL penicillin, and 100 *μ*g/mL streptomycin in a humidified incubator with 5% CO_2_ at 37°C.

### 2.6. Grouping and Experimental Design

Human breast cancer nude mouse xenograft model was established by injecting MDA-MB-231 cell suspension (1 × 10^7^ cells/mL) from the left second pair breast pad in a volume of 0.5 mL each. Consequently, the oval skin bulge can be seen, with the nipple located at the center of the bulge. Tumors (visualized as a small nodule with an approximate size of 20–30 mm^3^ at the site of injection) appeared approximately 5–7 days after the injection. The mice were randomly and equally divided into three groups according to body weight and tumor size: triple-negative breast cancer control group (CON), tetrandrine group (TET), and capecitabine positive control group (Cap). The Cap group was intragastrically administered 0.1 mL of 10 mg/mL capecitabine solution, and the TET group was given 0.1 mL of 20 mg/mL TET solution, while the CON group was administered an equivalent volume of distilled water for 4 weeks.

### 2.7. Tumor Measurement

The tumor growth and body weight of the mice were monitored on the 6^th^, 12^th^, and 24^th^ day after intervention. The tumor volume was measured weekly by calipers until the mice were sacrificed under anesthesia. Each tumor was excised and weighed after the mice were sacrificed on day 29.

### 2.8. Sample Collection

After 4-week intervention, the mice were fasted for 24 h, followed by sacrificing with 1% pentobarbital sodium (0.1 mg/g). The tumor tissue was excised and stripped. The tumor weight and volume were measured, after which one section of the tumor tissue was fixed in 4% paraformaldehyde solution for histopathology and one section was frozen in liquid nitrogen for further analysis.

### 2.9. Morphological Observation

Fresh tumor tissue samples were fixed in 4% paraformaldehyde. Then, the fixed tissues were embedded in paraffin, cut into 5 *μ*m slices, and stained with hematoxylin and eosin (HE), according to the standard protocol. The stained sections were observed and images acquired under a light microscope at 50×, 100×, and 400× magnification.

### 2.10. Immunohistochemistry Analysis of Bcl-2-Associated X Protein (BAX) and B-Cell Lymphoma/Leukemia-2 (BCL-2)

The fresh tumor tissue samples were fixed in 4% paraformaldehyde and embedded in paraffin. 5 *μ*m thick paraffin sections were mounted on glass slides and treated with 3% hydrogen peroxide for 10 min at room temperature. After washing with phosphate-buffered saline (PBS), the sections were blocked with sheep serum for 10 min at room temperature, followed by incubation with anti-BAX or anti-BCL-2 primary antibodies at 4°C overnight. The secondary antibodies were applied for 30 min at room temperature, and, then, the slides were stained with diaminobenzidine (DAB) and hematoxylin. The immunohistochemistry staining was observed under a microscope (BX-53; Olympus).

### 2.11. RNA Isolation and RT-qPCR

Total RNA was extracted using TRIzol reagent according to the manufacturer's instructions. The concentration and purity of the RNA samples were measured. cDNA was synthesized for reverse transcription-polymerase chain reaction (RT-PCR), according to the instructions in the First Strand cDNA Synthesis Kit (Invitrogen). The sequences of the primers used for the RT-PCR assay are shown in Table 1. The reverse transcription conditions were as follows: 37°C for 15 min, followed by 85°C for 5 s for RT inactivation. The amplification was performed using the following conditions: 30 s at 95°C for denaturation, 5 s at 95°C for annealing, 40 s at 60°C for extension.

### 2.12. Western Blot Analysis

Frozen tumor tissues were homogenized in ice-cold RIPA lysis buffer (Beijing, China), and the extract was collected by centrifugation at 12000 rpm for 15 min at 4°C. The total protein concentration was determined by the bicinchoninic acid (BCA) protein assay kit (Beijing Pulilai Gene Technology Co., Ltd., Beijing, China). An equivalent amount of protein sample was separated by 12% sodium dodecyl sulfate polyacrylamide gel electrophoresis (SDS-PAGE) and transferred to a polyvinylidene difluoride (PVDF) membrane (Millipore, USA). Then, the membranes were blocked at room temperature with 5% dried skimmed milk for 1 h and incubated at 4°C overnight with primary antibodies: Caspase-3, Survivin, Bax, Bcl-2, Bid, PARP, and *β*-actin. The washed membranes were incubated with the corresponding secondary antibodies for 1 h at room temperature. Finally, the immunoreactive bands were detected using an ECL detection system (Tanon, Shanghai, China) and quantified using Image J software.

### 2.13. Statistical Analysis

Statistical analysis was performed using SPSS 17.0 statistical software (IBM, Armonk, NY, USA). Data were presented as mean ± standard deviation (SD). Significant differences among groups were analyzed by one-way analysis of variance (ANOVA), followed by least-square difference test. *P* < 0.05 was considered statistically significant.

## 3. Results

### 3.1. Effect of TET on Body Weight

With the experiment prolonged duration, compared to the control group, the other groups of nude mice showed weight loss, slow response, arched neck, and prominent spine. The body weights of nude mice at different time points are shown in Table 2.

### 3.2. Effect on Tumor Weight and Volume

The tumor volume of each group of nude mice increased with time, establishing a positive correlation between the two parameters.

After 24-day intervention, the tumor weight in the TET and Cap groups decreased significantly as compared to the control group (*P* < 0.05). Compared to the TET group, the tumor weight in the Cap group decreased significantly (*P* < 0.05) (Table 3).

**Figure 1:**
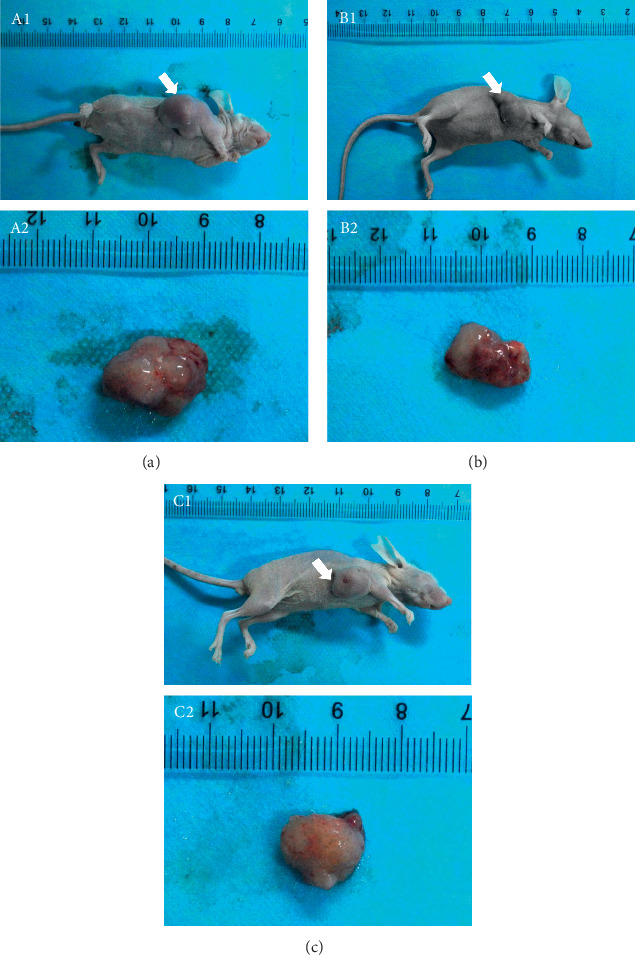
Antitumor activity of TET and Cap in MDA-MB-231 mouse xenografts. Photographs of tumor appearance (A1: control group; B1: TET group; C1: Cap group). Photographs of isolated tumors derived from control and treated mice (A2: control group; B2: TET group; C2: Cap group).

Compared to the control group, tumor weight and volume in both TET and Cap groups decreased significantly (*P* < 0.05). Compared to the TET group, the tumor weight and volume in the Cap group decreased significantly (*P* < 0.05; Table 4, Figure 1).

### 3.3. Histopathological Changes in the Tumor Tissue

**Figure 2:**
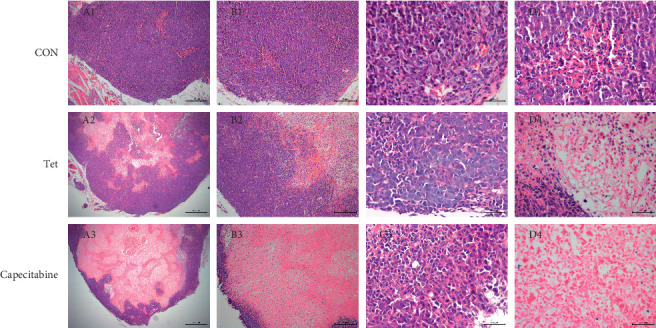
Morphological changes of tumor tissue in the CON, TET, and cap groups. A represents tumor full-section at 50× magnification; B represents tumor junction-section at 100× magnification; C represents tumor marginal area at 400× magnification; and D represents tumor core region at 400× magnification.

The cells of tumor tissues in all the groups were disorderly arranged and nested, with different morphologies and enlarged nuclei. Compared to the control group, different levels of inflammatory cell aggregates were distributed in a sheet form in the TET and Cap groups. In the Cap group, the nuclei were almost invisible and stained with pink, indicating cell rupture and cytoplasmic outflow. In the TET group, different degrees of pink cytoplasmic staining and mixed purple-blue nuclear staining were observed. Compared to the control group, the pink cytoplasm staining area in both TET and Cap groups was larger, indicating a high proportion of cell necrosis (Figure 2).

### 3.4. Immunohistochemistry Analysis of Bcl-2 and Bax

Compared to the control group, the expression of Bcl-2 decreased significantly in the TET and Cap groups. The decreased expression of Bcl-2 in the Cap group was superior to that in the TET group.

**Figure 3:**
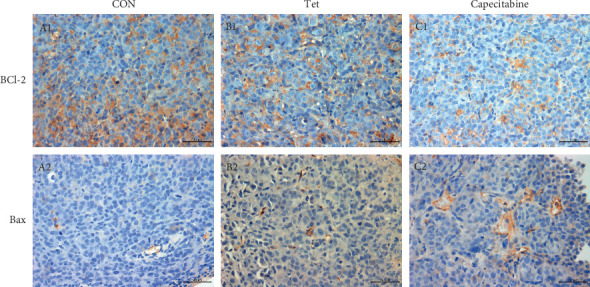
TET induces cell apoptosis in TNBC cell line MDA-MB-231 xenografts. A represents CON group; B represents TET group; and C represents cap group. The expression of Bcl-2 and Bax in tumors was detected by immunohistochemistry (400× magnification).

Compared to the control group, the expression of Bax was increased in both TET and Cap groups; interestingly, the increased expression in the Cap group was superior to that in the TET group (Figure 3).

### 3.5. Gene Expression

**Figure 4:**
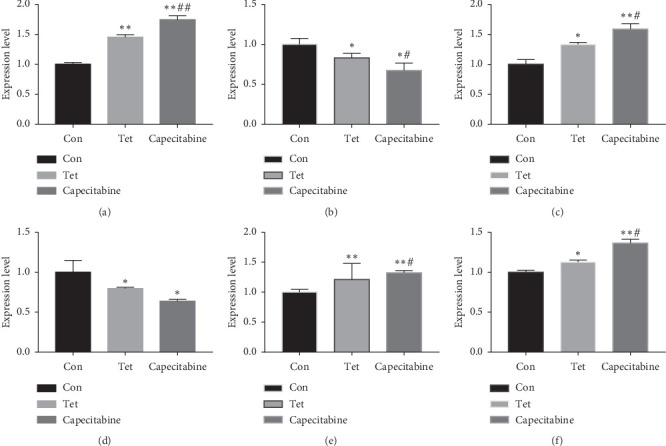
Effect of TET on apoptosis-associated gene expression in the breast tumor as determined by RT-qPCR. The levels of (a) Caspase-3, (b) *Survivin*, (c) *Bax*, (d) *Bcl-2*, (e) *Bid*, and (f) *PARP* genes in tumors were determined by RT-qPCR. ^*∗*^Compared to the control group, *P* < 0.05; ^*∗∗*^compared to the control group, *P* < 0.01; ^#^compared to the TET group, *P* < 0.05; ^##^compared to the TET group, *P* < 0.01.

Compared to the control group, the expression of Caspase-3, Bax, Bid, and PARP increased significantly in the TET and Cap groups (*P* < 0.05, 0.01), while the expression of Bcl-2 and Survivin in the TET and Cap groups decreased significantly (*P* < 0.05). Compared to the TET group, the expression of Caspase-3, Bax, Bid, and PARP increased significantly in the Cap group (*P* < 0.05, 0.01), while the expression of Survivin decreased significantly (*P* < 0.05) (Figure 4).

### 3.6. Protein Expression

**Figure 5:**
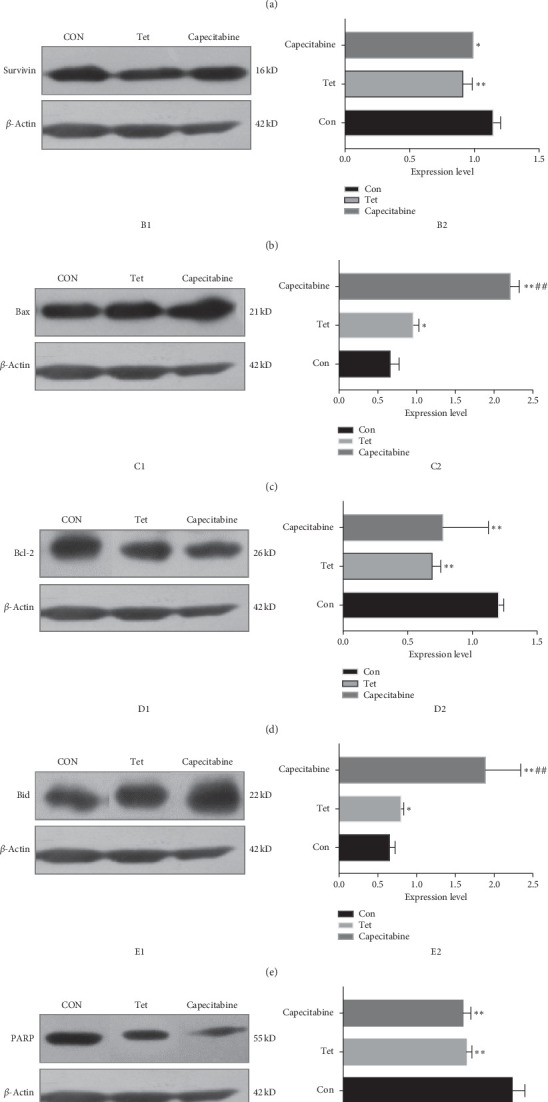
Effect of TET on apoptosis-associated protein expression in the breast tumor as determined by Western blotting. The protein levels of (a) Caspase-3, (b) Survivin, (c) Bax, (d) Bcl-2, (e) Bid, and (f) PARP in tumors were determined by Western blotting. A represents expression of caspase 3; B represents expression of Survivin; C represents expression of Bax; D represents expression of Bcl-2; E represents expression of Bid; F represents expression of PARP. ^*∗*^Compared to the control group, *P* < 0.05; ^*∗∗*^compared to the control group, *P* < 0.01; ^##^compared to the TET group, *P* < 0.01.

Compared to the control group, the expression of Caspase-3, Bax, and Bid proteins increased significantly in both TET and Cap groups (*P* < 0.05, 0.01), while that of Bcl-2, Survivin, and PARP decreased significantly in both TET and Cap groups (*P* < 0.05, 0.01). Compared to the TET group, the protein expression of Caspase-3, Bax, and Bid proteins increased significantly in the Cap group (*P* < 0.01) (Figure 5).

## 4. Discussion

In this study, the anticancer activity of TET against the TNBC cell line MDA-MB-231 in vivo was investigated by focusing on apoptosis. In addition to morphological change, the expression of apoptosis-associated genes, such as *Caspase-3*, *Survivin*, *Bax*, *Bcl-2*, *Bid*, and *PARP* was detected.

The present study demonstrated that TET markedly inhibits the growth of tumors in MDA-MB-231 mouse xenografts, as the tumor weight and volume decreased significantly in the TET and Cap groups. This finding was consistent with that of Zhang et al., who demonstrated that TET effectively suppressed the tumor growth in cervical cancer [25]. The morphological analysis demonstrated that the tumor cells were arranged in a disorderly, irregular, nested shape, with different sizes and shapes, and enlarged nucleus, as observed in the TNBC control group. In TET and Cap groups, inflammatory cells accumulated due to cell necrosis. These results suggested that TET exerted notable antitumor effects on TNBC cell line MDA-MB-231 in vivo.

Apoptosis plays a major role in killing tumor cells [25]. It is a gene-regulated cell death process, also known as programmed cell death, which occurs in all living cells and is regulated by genes, characterized by a series of cellular morphological changes, including chromosome condensation, nuclear fragmentation, cell shrinkage, and the formation of apoptosome [21]. The extrinsic and intrinsic signaling pathways lead to apoptosis. The extrinsic pathway provokes apoptosis through a caspase cascade that eventually leads to cell death, while the intrinsic apoptotic pathway is mitochondria-dependent and responds to different stress conditions, such as cytosolic calcium, genetic damage, and oxidative stress [33]. Both pathways triggered caspase cascade, converged on Caspase-3, and ultimately led to the morphological characteristics of apoptosis [24].

The Bcl-2 family is involved in the regulation of cell apoptosis, including a series of antiapoptotic and proapoptotic members. The Bcl-2 apoptotic signaling pathway activates the apoptotic pathway through multiple intercellular and intracellular signals. Within the Bcl family, Bax is a proapoptotic protein, which promotes apoptosis by activating caspases. Conversely, Bcl-2 is an antiapoptotic protein, which prevents apoptosis by inhibiting the release of mitochondrial apoptogenic factors into the cytoplasm. Bcl-2 and Bax are typical proteins that inhibit and promote apoptosis, respectively, in this pathway. Apoptosis is mainly determined by the relative concentration of Bcl-2 and Bax. An imbalance of Bax and Bcl-2 proteins may lead to the loss of MMP and the release of cytochrome C, which activates Caspase-3 and results in apoptosis [21, 34–37]. The BID protein is a member of another group of the Bcl-2 family. It activates apoptosis as well as integrating two main apoptotic routes, connecting the membranous (external) and mitochondrial (internal) pathways [38]. In this study, the immunohistochemistry analysis showed decreased expression of Bcl-2 in the TET and Cap groups as compared to the CON group; however, the decreased expression of Bcl-2 in the Cap group was superior to that in the TET group. Compared to the control group, the expression of Bax was increased in both the TET and Cap groups, while the increased expression in the Cap group was superior to that in the TET group. RT-PCR and Western blotting results showed that Tet decreased the level of antiapoptotic gene and protein Bcl-2 and increased the level of proapoptotic genes and proteins, Bax and Bid. Considering that these three proteins are closely associated with mitochondria, it could be speculated that Tet induced apoptosis through mitochondrial disruption by changing the levels of the apoptosis-associated proteins, Bcl-2, Bax, and Bid.

Caspase-3 plays a major role in triggering the apoptotic process, and its activity has been suggested to be an index of apoptosis, which is commonly considered as the most important terminal cleavage enzyme during apoptosis. PARP is involved in DNA repair and transcriptional regulation and is regarded as a critical regulatory factor of cell survival and cell death. It also participates in the regulation of transcription factors in tumorigenesis and inflammatory response [39]. During apoptosis, PARP is cleaved by Caspase-3 into two fragments of 31 kDa and 85 kDa. This cleavage makes PARP unable to function normally, causing apoptosis [40]. Survivin belongs to the inhibitor of apoptosis protein (IAP) family and is potentially involved in both facilitating tumor cell proliferation and inhibiting apoptosis. It is lowly expressed in normal cells, but highly during tumor proliferation and angiogenesis. Tumor apoptosis can be induced by inhibiting this protein [41]. It has been shown that Survivin can directly block the process and activation of the cell death terminal effector Caspase-3 and induce the apoptosis of cells [37]. In this study, compared to the control group, the expression of Caspase-3 gene and protein increased significantly in both TET and Cap groups, while that of Survivin and PARP decreased significantly in both groups. These results demonstrated that TET induces apoptosis by the activation of Caspase-3, cleavage of PARP, and downregulation of Survivin in TNBC in vivo.

## 5. Conclusion

In this study, we demonstrated that TET effectively inhibits tumor growth and induces apoptosis in TNBC cell line MDA-MB-231. The mechanism underlying this effect may be mediated by TET-upregulated Caspase-3, Bax, and Bid, and downregulated Bcl-2, Survivin, and PARP. This study showed that TET is a promising therapeutic agent for the treatment of TNBC. Considering TNBC is one of the most difficult subtypes of breast cancer to treat due to its aggressive, metastatic behavior and a lack of targeted therapy, further studies about the effect of TET on the chemotherapeutic resistance are imperative.

## Figures and Tables

**Table 1:** Primer sequences of RT-qPCR.

Gene	Upstream primer	Downstream primer
*Caspase-3*	5′-GGAGGCCGACTTCTTGTATG-3′	5′-ACTGTTTCAGCATGGCACAA-3′
*Bid*	5′-GGCCTACCCTAGAGACATGGA-3′	5′-AGACATCACGGAGCAAGGAC-3′
*Bax*	5′-ATGGGCTGGACATTGGAC-3′	5′-GGGACATCAGTCGCTTCAG-3′
*Bcl-2*	5′-ATGTGTGTGGAGAGCGTCAA-3′	5′-GAGACAGCCAGGAGAAATCAA-3′
*Survivin*	5′-GGACCACCGCATCTCTACAT-3′	5′-CAAGTCTGGCTCGTTCTCAGT-3′
*PARP*	5′-CCGCATACTCCATCCTCAGT-3′	5′-GCTTCTTCATCCCAAAGTCG-3′
*GAPDH*	5′-AGAAGGCTGGGGCTCATTTG-3′	5′-AGGGGCCATCCACAGTCTTC-3′

**Table 2:** Body weight of nude mice at different time points (mean ± SD).

Groups	*N*	Day 0 (g)	Day 6 (g)	Day 12 (g)	Day 24 (g)
CON	10	14.56 ± 0.33	15.47 ± 0.26	16.92 ± 0.12	18.02 ± 0.27
TET	10	14.64 ± 0.45	15.06 ± 0.12	15.13 ± 0.21^*∗*^	15.28 ± 0.30^*∗*^
Cap	10	14.53 ± 0.45	15.15 ± 0.26	15.13 ± 0.20^*∗*^	15.47 ± 0.50^*∗*^

Note: ^*∗*^compared to the control group, *P* < 0.05.

**Table 3:** Tumor volume at different time points (mean ± SD) (*n* = 10).

Groups	*N*	D0 (cm^3^)	D6 (cm^3^)	D12 (cm^3^)	D24 (cm^3^)
CON	10	0.04 ± 0.01	0.84 ± 0.06	1.57 ± 0.03	2.50 ± 0.02
TET	10	0.04 ± 0.01	0.77 ± 0.05	1.33 ± 0.04^*∗*^	1.85 ± 0.04^*∗*^
Cap	10	0.04 ± 0.01	0.69 ± 0.02^*∗*^	1.36 ± 0.03^*∗*^	1.37 ± 0.03^*∗*#^

Note: ^*∗*^compared to the control group, *P* < 0.05; ^#^compared to the TET group, *P* < 0.05.

**Table 4:** Tumor weight and volume after excision (mean ± SD) (*n* = 10).

Groups	*N*	Weight (g)	Volume (cm^3^)
CON	10	3.28 ± 0.05	2.62 ± 0.03
TET	10	2.65 ± 0.03^*∗*^	2.01 ± 0.03^*∗*^
Cap	10	1.92 ± 0.04^*∗*#^	1.44 ± 0.03^*∗*#^

Note: ^*∗*^compared to the control group, *P* < 0.05; ^#^compared to the TET group, *P* < 0.05.

## Data Availability

The data supporting the conclusions of this study are available to all interested readers upon request to the corresponding author (pxh_127@163.com).
